# Contrast enhanced ultrasound - a useful method for diagnosing tubal ectopic pregnancy with low level β-HCG

**DOI:** 10.1186/s13089-025-00438-0

**Published:** 2025-07-23

**Authors:** Jingping Wu, Xinyu Chen, Qiong Wu, Na Wei, Guifang An, Hairong Yu, Ning Wang

**Affiliations:** 1 Department of Ultrasound Medicine, China–Japan Friendship Hospital, 2 Yinghua East Street, Chaoyang District, Beijing, 100029 China; 2 Department of Ultrasound, Dongzhimen Hospital of Beijing University of Chinese Medicine, 5 Haiyun Cang, Dongcheng District, Beijing, 100700 China

**Keywords:** Ultrasonography, Contrast media, Pregnancy, Ectopic, Fallopian tubes

## Abstract

**Background:**

In patients presenting with atypical symptoms or low plasma β-HCG levels, the presence of an adnexal mass without a yolk sac or embryo on ultrasound often provides insufficient confidence for a definitive diagnosis of ectopic pregnancy(EP). Consequently, most such cases can only be classified as suspected EP. This study aimed to evaluate the diagnostic value of contrast enhanced ultrasound (CEUS) for tubal EP (tEP). We retrospectively analyzed 21 patients with suspected EP who underwent CEUS between August 2017 and August 2024. All patients had plasma β-HCG levels below 3500 mIU/mL. Among them, 20 underwent laparoscopic surgery, and all underwent curettage. The diagnostic performance of CEUS for tEP was assessed.

**Results:**

A total of 21 patients were included: 19 with tEP, 1 with ovarian pregnancy, and 1 with intrauterine pregnancy. The sensitivity, specificity, and accuracy of transvaginal ultrasound (TVUS) for diagnosing tubal dilation were 15.8%, 100%, and 23.8%, respectively. For CEUS, these values were 94.7%, 100%, and 95.2%, respectively. Statistically significant differences were observed between CEUS and TVUS in sensitivity and accuracy (*P* = 0.000). The enhancement pattern of villous tissue was categorized as either circular or non-circular. Among the tEP cases, 2 exhibited circular enhancement and 17 showed non-circular enhancement. Based on positive β-HCG, absence of an intrauterine gestational sac, and sonographic visualization of a dilated fallopian tube containing either circular or non-circular enhancement internally, CEUS demonstrated high diagnostic accuracy for tEP diagnosis in cases with low β-HCG levels. CEUS correctly diagnosed 18 of 19 tEP cases. One tEP case was diagnosed as a mass of uncertain significance. The intrauterine pregnancy case was misdiagnosed as an EP. The ovarian pregnancy case was diagnosed as EP, though CEUS indicated a relatively high possibility of ovarian origin.

**Conclusion:**

In conclusion, CEUS holds significant diagnostic value for tEP. It is particularly useful in diagnostically unclear cases and provides a more detailed assessment of the internal structure of adnexal masses.

## Background

Ectopic pregnancy(EP) occurs in 1–2% of all pregnancies and can cause significant intra-abdominal bleeding, shock, and even death in pregnant women [1, 2]. The mortality rate of EP is approximately 3.6 per 10,000 cases in the United Kingdom, while in developing countries this number can be doubled [3].Between 1980 and 2007, 876 deaths were attributed to EP in the United States and the EP mortality ratio declined from 1.15 to 0.50 deaths per 100,000 live births between 1980 and 1984 and 2003–2007 [4].EP is the main cause of first-trimester mortality, accounting for approximately 4% of all pregnancy related deaths [5].

The most common ectopic implantation site is the fallopian tubes, which accounts for 95 − 98% of EP, and other implantation sites include ovaries, cervix, abdominal cavity, and cesarean section scars [6]. Therefore, early diagnosis of tEP is of great value for the patients.

Many guidelines and related literatures indicate that transvaginal ultrasound(TVUS) is the most important tool in diagnosing EP [7–15].According to previous literature, an important criterion for diagnosing tEP is a mass in the adnexal region that moves separate to the ovary [7].However, a consensus on nomenclature, definitions, and outcome of women initially classified as pregnancy of unknown location, proposes that an EP can definitely be diagnosed only when an extrauterine gestational sac with a yolk sac and/or embryo (with or without cardiac activity) is visualized [16]. However, the probability of occurrence of this ultrasound signs is relatively low in EP patients.In Condous G’s research, the majority of confirmed EP were seen as an inhomogeneous mass or blob sign (57.9%),20.4% were visualized as a hyper-echoic ring or bagel sign and only 13.2% were visualized as gestational sac with a fetal pole — 55% had positive fetal cardiac activity and 45% had no fetal cardiac activity [11]. According to the consensus, a diagnose of probable EP can be made if an inhomogeneous adnexal mass or a hyper-echoic ring is found by ultrasound [16], that is, most patients can only be diagnosed with suspected EP. Further more, the positive predictive value of those ultrasonic signs is only 80% because these findings can be confused with pelvic structures, such as a paratubal cyst, corpus luteum, hydrosalpinx, endometrioma, or bowel [17].

The “discriminatory level” means that there is a human chorionic gonadotropin(HCG) value above which the landmarks of a normal intrauterine gestation should be visible on a TVUS in a normal intrauterine pregnancy [18–20]. If “discriminatory level” is used to diagnose EP, in order to avoid misdiagnosis, some studies suggest setting the diagnostic threshold at 3500 mIU/mL [18, 20]. However, the HCG levels of EP patients vary greatly, often below 1000 mIU/mL and low level of HCG does not rule out the possibility of EP rupture [21, 22]. Barnhart KT et al. pointed out in their study that sensitivity of TVUS diagnosis of intrauterine pregnancy, spontaneous miscarriage, and EP in women who presented with β-HCG levels below 1500 mIU/mL was 33.3%, 28.2%, and 25.0%, respectively [23]. So, in clinical practice, for patients with atypical symptoms or low HCG levels, the confidence of doctors in diagnosing EP is insufficient when only an adnexal mass without a yolk sac or embryo appears. Therefore, a more definitive diagnostic method is needed in clinical work.Some literature reports the application of CT and MRI in the diagnosis of EP [24, 25]. However, the low soft tissue resolution and excessively expensive value limit the application of CT and MRI in the diagnosis of EP.There are few reports on the use of contrast enhanced ultrasound(CEUS)in the diagnosis of EP [26–29].These documents are mostly about cervical pregnancy or scar pregnancy.There is only one literature on tEP published by our team [30], However, no specific analysis was conducted on EP patients with low serum HCG levels in the aforementioned studies. This significant knowledge gap motivated our retrospective study.

## Methods

### Study population

We retrospectively analyzed 224 patients with suspected EP who underwent TVUS between August 2017 and August 2024. This study was approved by the ethics committee of Dongzhimen Hospital of Beijing University of Chinese Medicine(2024DZMEC-665-02).Our hospital is a third-level comprehensive hospital (the highest-level hospitals in China).The informed consent of patients is waived because this is a retrospective analysis.

The inclusion criteria were as follows: **(a)** Patients were diagnosed with suspicious EP, including the following characteristics: the patient’s HCG level exceeds the normal value; a mass in the adnexal region was found in patients; patients have the following symptoms such as abdominal pain, delayed menstruation, vaginal bleeding. **(b)** Patients underwent preoperative CEUS and TVUS. **(c)** Patients had no pregnancy plan at the time of examination and agreed to undergo CEUS. **(d)** Patients’ vital signs were stable, that is, there were no signs of decreased blood pressure or shock.**(e)** The β-HCG level in plasma of patients was lower than 3500 mIU/mL and the time interval between CEUS and β-HCG detection was less than 24 h, or β-HCG level in plasma was lower than 500 mIU/mL and the time interval between CEUS and β-HCG detection was 24 to 48 h.

The exclusion criteria are as follows: **(a)** Patients did not undergo surgical treatment and had no pathological results. **(b)** Patients refused CEUS. **(c)** Patients did not meet the indications for CEUS (age less than or equal to 18 years old; history of contrast agent allergy). **(d)** EP can be definitively diagnosed by TVUS(extrauterine gestational sac with a yolk sac and/or embryo with or without cardiac activity was visualized). **(f)** Related clinical data was incomplete.

**Fig. 1:**
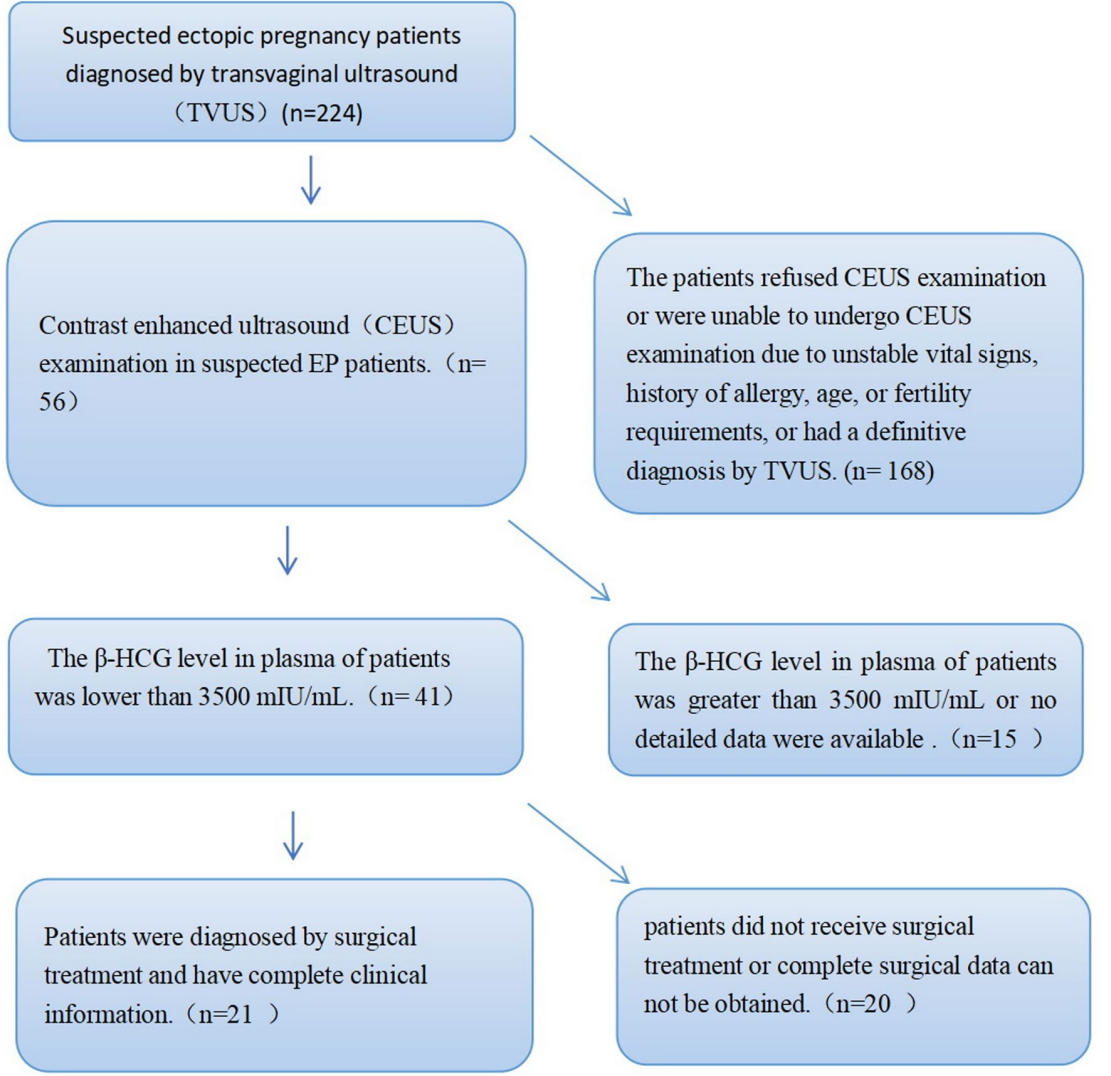
A flow diagram of patient enrollment process

Finally, a total of 21 patients were included in the study **(**Fig. 1**)**. The final diagnoses of all the patients are as follows: 19 cases of tEP, 1 case of ovarian pregnancy, and 1 case of intrauterine pregnancy. Among these patients, 20 underwent laparoscopic surgery, and all patients underwent curettage.

### TVUS and CEUS

During TVUS, we used the GE Logiq E9 color Doppler ultrasound diagnostic instrument with a probe frequency of 4–9 MHz and a mechanical index (MI) of 0.06–0.08. The routine examination steps of TVUS were as follows: Firstly, we conducted a detailed examination of the patient’s uterus, focusing on observing whether there was a gestational sac in the uterine cavity, while also observing the morphology and structure of the uterus; Secondly, both ovaries were scanned. Thirdly, both fallopian tubes and the pelvic cavity were observed to find any abnormal echoes, with a particular focus on the painful areas of the patient.Referring to relevant literature [11], if an inhomogeneous mass was found in the adnexal area, we defined it as a blob sign. If a mass with a hyper-echoic ring was found in the adnexal area, we defined it as a bagel sign.

CEUS was generally performed after TVUS.Due to the prohibition of using ultrasound contrast agents on pregnant women, we strictly excluded patients who had pregnancy plan at the time of examination.The instruments and probes used for CEUS were the same as those used for TVUS. The contrast agent was SonoVue (Bracco corp., Milan, Italy). Routine process of CEUS was as follows. Firstly, the contrast agent was diluted with 5 ml of physiological saline to form a microbubble suspension; secondly,2.4 ml suspension was extracted and injected into the patient’s body via the elbow vein and 5.0 ml of physiological saline was used for flushing; thirdly, the enhancement of adnexal masses were being carefully observed. It should be noted that during the examination, the probe needs to be moved to observe the overall condition of the mass, rather than just one ultrasound section.If a tubular structure was found, the whole tubular structure should be fan-shaped scanned to determine the size, morphology, enhancement characteristics; if there was a enhancement of villous structure in the tubular structure, its size, morphology, enhancement characteristics, relationship with tubular structure should be carefully observed. The observation time of first injection was 3 minutes. Fourthly, 2.4 milliliters of contrast agent was reinjected to observe the enhancement of the mass for the second time.The observation time of second injection was 3 minutes too. Depending on whether there was enhancement, we divided the pattern of soft tissue enhancement into with enhancement and without enhancement.Regarding the enhancement pattern of intra-tubal villous tissue, we defined as follow: circular enhancement was characterized by enhancement of a ring-like structure with an inner area of no enhancement and a ring wall thickness of > 3 mm. non-circular enhancement was characterized by any presentation other than circular enhancement.

TVUS and CEUS examinations of 21 patients were completed by 6 different doctors. Among them, 2 doctors had more than 20 years experience in ultrasound examination, and the remaining 4 doctors had 6–10 years of experience in ultrasound examination. All doctors have received standardized training in ultrasound medicine.In this study, the diagnosis of TVUS and CEUS of all patients was completed by an ultrasound specialist with more than 20 years of work experience.

### Clinical data

Most of clinical data (such as age, HCG levels, surgical records, etc.) were obtained from Dongzhimen Hospital’s Picture Archiving and Communication Systems.We obtained relevant medical records through follow-up for patients who underwent surgery in other hospitals.

### Study design

Our research has found that CEUS can effectively identify the structure of fallopian tubes and the villous. Firstly, we analyzed the differences in the recognition of fallopian tubes between CEUS and TVUS; secondly, CEUS manifestations and classification of villous tissue in suspicious EP patients with low level β-HCG were studied; thirdly, the value of CEUS in diagnosing tEP was analysed.

### Statistical analysis

Statistical analysis was performed using SPSS version 24.0 (IBM Corporation) for Windows.Descriptive results were expressed as mean with standard deviation. The chi square test or Fisher’s exact test was applied to compare two sets of data rates, such as sensitivity, specificity, and accuracy.Differences with *P* < 0.05 were considered to be statistically significant.

## Results

### Patient characteristics

The median age of all patients was 31 years, and the range was 23–41 years; Median value of the maximum diameter of the mass was 3.2 cm, and the range was 1.5–8.9 cm.The median HCG level in the blood was 633mIU/mL, with a range of 84-3137mIU/mL. Table 1 showed the detailed information of all patients. Among 20 EP cases, TVUS manifested as blob sign in the adnexal area in 18 cases and as bagel sign in 3 cases(One patient showed two signs simultaneously.).

**Table 1:** Clinical and imaging manifestations of all patients

	Age(years)	HCG (mIU/mL)	Mass size (cm)	Blob sign (TVUS)	Circular enhancement of villous tissue(CEUS)	Bagel sign (TVUS)	Dilated tubular structures visible on CEUS	Dilated suspicious tubular structures visible on TVUS	Rupture of the fallopian tubes	Final diagnosis
1	28	84	2.2 × 2.0	No	No	No	No	No	No	**IUP**
2	25	201	5.9 × 2.7	Yes	No	No	Yes	No	No	Right tEP
3	28	319	3.1 × 2.7	Yes	No	No	Yes	No	No	Left tEP
4	24	320	3.2 × 2.7	Yes	No	No	Yes	No	No	Right tEP
5	40	333	8.9 × 6.4	Yes	No	No	Yes	No	No	Left tEP
6	31	350	2.4 × 1.0	Yes	No	No	Yes	No	No	Left tEP
7	27	381	6.3 × 4.5	Yes	No	No	Yes	No	No	Right tEP
8	37	406	6.3 × 6.1	Yes	No	No	No	No	Yes	Left tEP
9	26	484	3.2 × 2.2	Yes	No	No	Yes	No	No	Right tEP
10	37	498	2.9 × 1.6	Yes	No	No	Yes	No	No	Right tEP
11	30	633	4.3 × 2.4	Yes	No	No	Yes	No	No	Left tEP
12	31	714	4.2 × 1.5	Yes	No	No	Yes	Yes	No	Left tEP
13	39	806	3.6 × 1.8	Yes	No	No	Yes	No	No	Right tEP
14	30	929	3.2 × 1.3	**Yes**	**Yes**	**Yes**	Yes	No	No	Left tEP
15	23	1010	2.4 × 2.1	Yes	No	No	No	No	No	Right **OP**
16	31	1113	5.5 × 1.8	Yes	No	No	Yes	Yes	No	Left tEP
17	33	1196	6.6 × 3.2	Yes	No	No	Yes	No	No	Right tEP
18	41	1834	3.2 × 2.7	Yes	No	No	Yes	No	No	Right tEP
19	36	1906	1.5 × 1.4	**No**	**Yes**	**Yes**	Yes	Yes	No	Right tEP
20	34	2165	1.7 × 1.4	**No**	**No**	**Yes**	Yes	No	No	Left tEP
21	25	3137	2.7 × 1.7	Yes	No	No	Yes	No	No	Right tEP

CEUS: Contrast enhanced ultrasound; TVUS: transvaginal ultrasound; tEP: tubal ectopic pregnancy; OP: ovarian pregnancy; IUP: Intrauterine pregnancy

### The value of TVUS and CEUS in identifying tubal dilation in suspicious EP patients with low level β-HCG (Table 1; Figs. 2, 3 and 4)

**Fig. 2:**
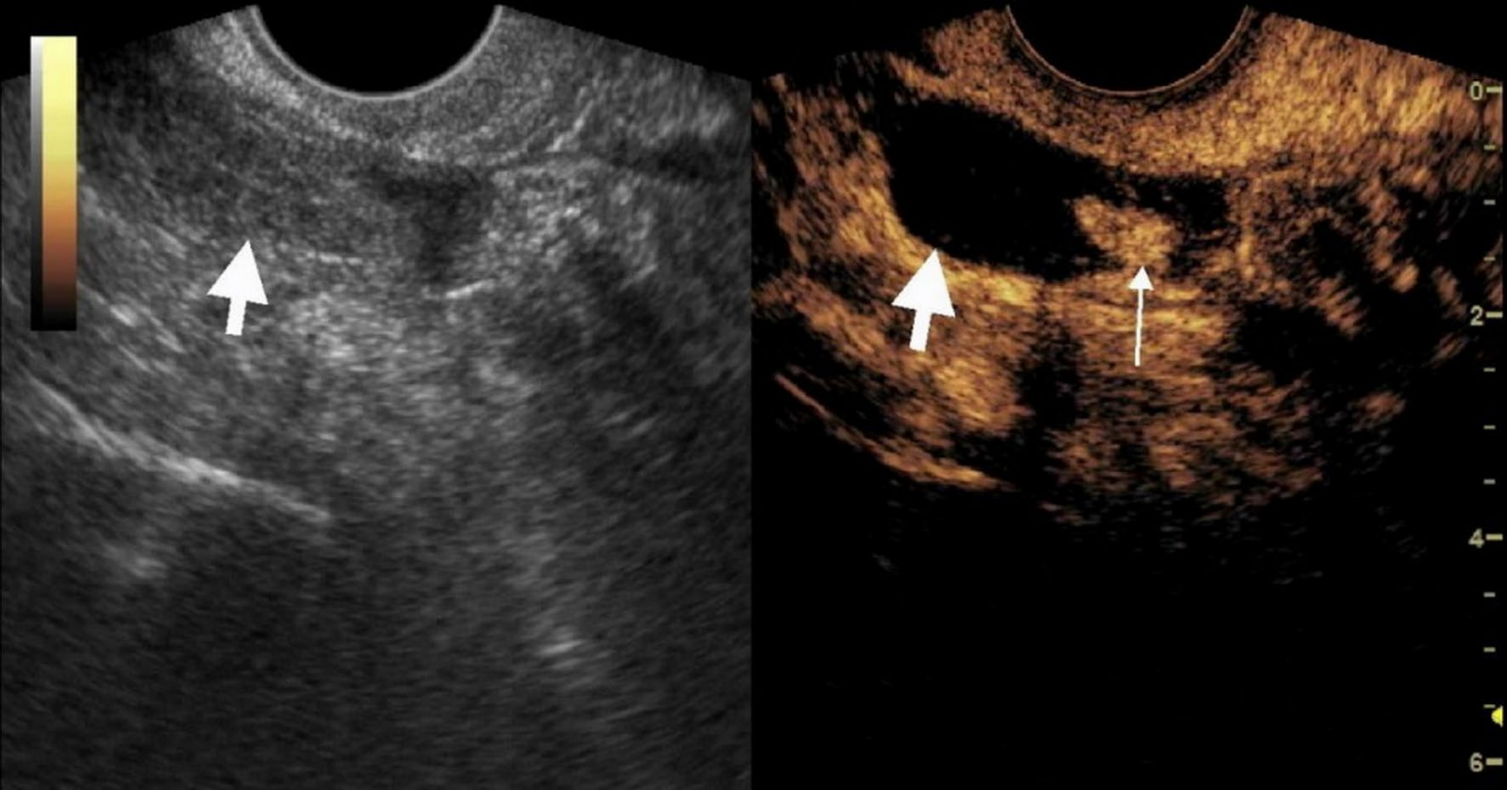
Transvaginal contrast enhanced ultrasound(CEUS) showed the longitudinal section of the fallopian tube. The patient was a 39-year-old woman who came to the hospital for amenorrhea and vaginal bleeding. Her blood HCG level was 806 mIU/mL. The time interval between CEUS and blood HCG measurement was less than 6h. Ultrasound examination found a low to medium echoic mass measuring 3.6cm × 1.8cm in the right adnexal region (left figure). The CEUS showed heterogeneous enhancement of the mass (right figure). The thick arrow indicated the dilated fallopian tube, and the thin arrow indicated the enhanced villous tissue. The non-enhanced area within the mass was confirmed intraoperatively to be blood clots within the fallopian tube. The villous tissue size visible during the operation was 0.5cm × 0.5cm, similar to the size measured by ultrasound (0.8cm × 0.5cm). The enhancement pattern of this patient was non-annular enhancement.This patient was confirmed by surgery to have a pregnancy in the ampulla of the right fallopian tube. Transvaginal contrast enhanced ultrasound (CEUS) showed the longitudinal section of the fallopian tube

**Fig. 3:**
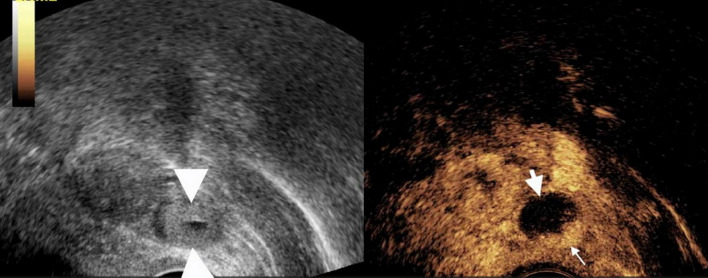
Transvaginal contrast enhanced ultrasound (CEUS) showed the cross section of the fallopian tube. The patient was 34 years old and came to the hospital due to abdominal pain, amenorrhea, and vaginal bleeding. The blood HCG level was 2165 mIU/mL, and the time interval between the CEUS and the measurement of blood HCG was less than 24 hours. A ring-like slightly high echoic structure (bagel sign) measuring 1.7cm × 1.4cm was visible in the left adnexal region, with a small area of anechoic in the center (left figure). CEUS imaging showed that, some parts of the high echoic ring-like structure enhanced, while other parts were not enhanced (right figure). The thick arrow indicated the dilated fallopian tube, and the thin arrow indicated the enhanced villous tissue. The villous tissue size visible during the operation was 0.5cm × 0.5cm, less than the size measured by ultrasound (0.8cm x 1.0cm).The enhancement pattern of this patient was non-annular enhancement. This patient was confirmed by surgery to have a pregnancy in the ampulla of the left fallopian tube

According to the findings during surgery, all cases of tEP showed the following characteristics: the fallopian tube was tortuous and dilated, appearing dark blue and inside it were dark red clots and villous tissue of various sizes. CEUS performance of the fallopian tube was characterized by an expanded tubular structure, with enhanced tubal walls and no enhancement in internal areas. The non-enhanced areas were confirmed postoperatively to be blood clots.Dilated fallopian tubes appear as long, low-echo structures on TVUS. Three cases were diagnosed by TVUS as suspicious dilated fallopian tubes.The sensitivity, specificity, and accuracy of TVUS and CEUS in diagnosing tubal dilation were 15.8%, 100%, 23.8%, and 94.7%, 100%, and 95.2%, respectively.There were statistically significant differences in sensitivity (Fisher’s exact test, *P* = 0.000)and accuracy (χ² test, *P* = 0.000) between CEUS and TVUS.

### CEUS manifestations and classification of villous tissue in suspicious EP patients with low level β-HCG (Table 1)

**Fig. 4:**
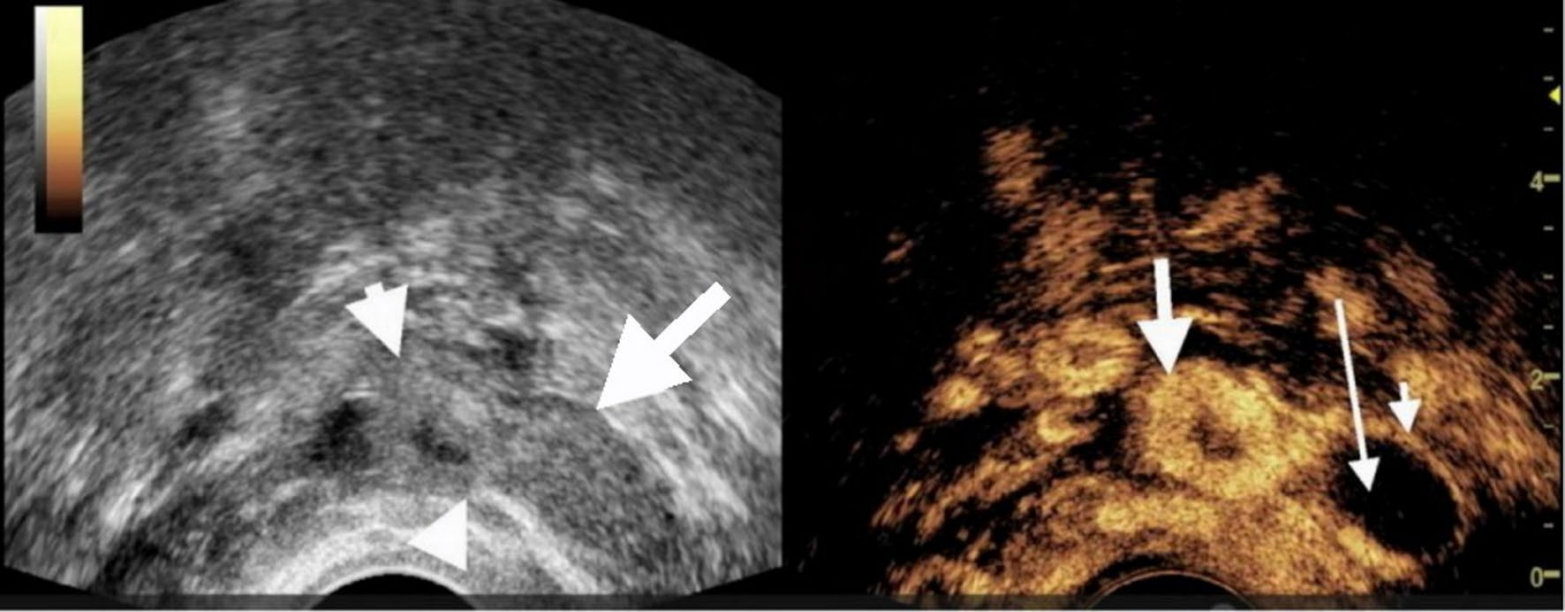
Transvaginal contrast enhanced ultrasound (CEUS) showed the longitudinal section of the fallopian tube. The patient was 30 years old and came to the hospital due to abdominal pain, amenorrhea, and vaginal bleeding. The blood HCG level was 929 mIU/mL, and the time interval between CEUS and the measurement of blood HCG was less than 6 hours. A mixed-echoic mass (blob sign) measuring 3.2cm× 1.3cm was seen in the left adnexal region with a portion of the mass demonstrating a bagel sign (arrows on the left and the middle of the left figure). CEUS showed heterogeneous enhancement of the mass. The area with the bagel sign demonstrated ring-like enhancement, while the other areas mainly showed tubular structures without enhancement. The thick arrow indicated the the enhanced villous tissue, the thin short arrow indicated dilated fallopian tube, and the thin long arrow indicated blood clots within the fallopian tube. This patient was confirmed by surgery to have a pregnancy in the ampulla of the left fallopian tube.There was no record of the size of the villous tissue

By comparing CEUS with surgical findings, we found that the villous tissue inside the fallopian tube generally presents as a enhanced structure within the lumen and tightly adheres to the fallopian tube (Figs. 2, 3 and 4). Enhancement pattern of villous tissue was divided into two types: circular enhancement (Fig. 4) and non-circular enhancement (Figs. 2 and 3). Among the cases of tEP, there were a total of 2 cases with circular enhancement and 17 cases with non circular enhancement of their villous tissues.The plasma HCG levels in the two patients with circular enhancement were 929 mIU/mL and 1906 mIU/mL respectively; in the non- circular enhancement group (17 patients), the HCG levels ranged from 201 to 3137 mIU/mL, a median value of 498 mIU/mL.

**Fig. 5:**
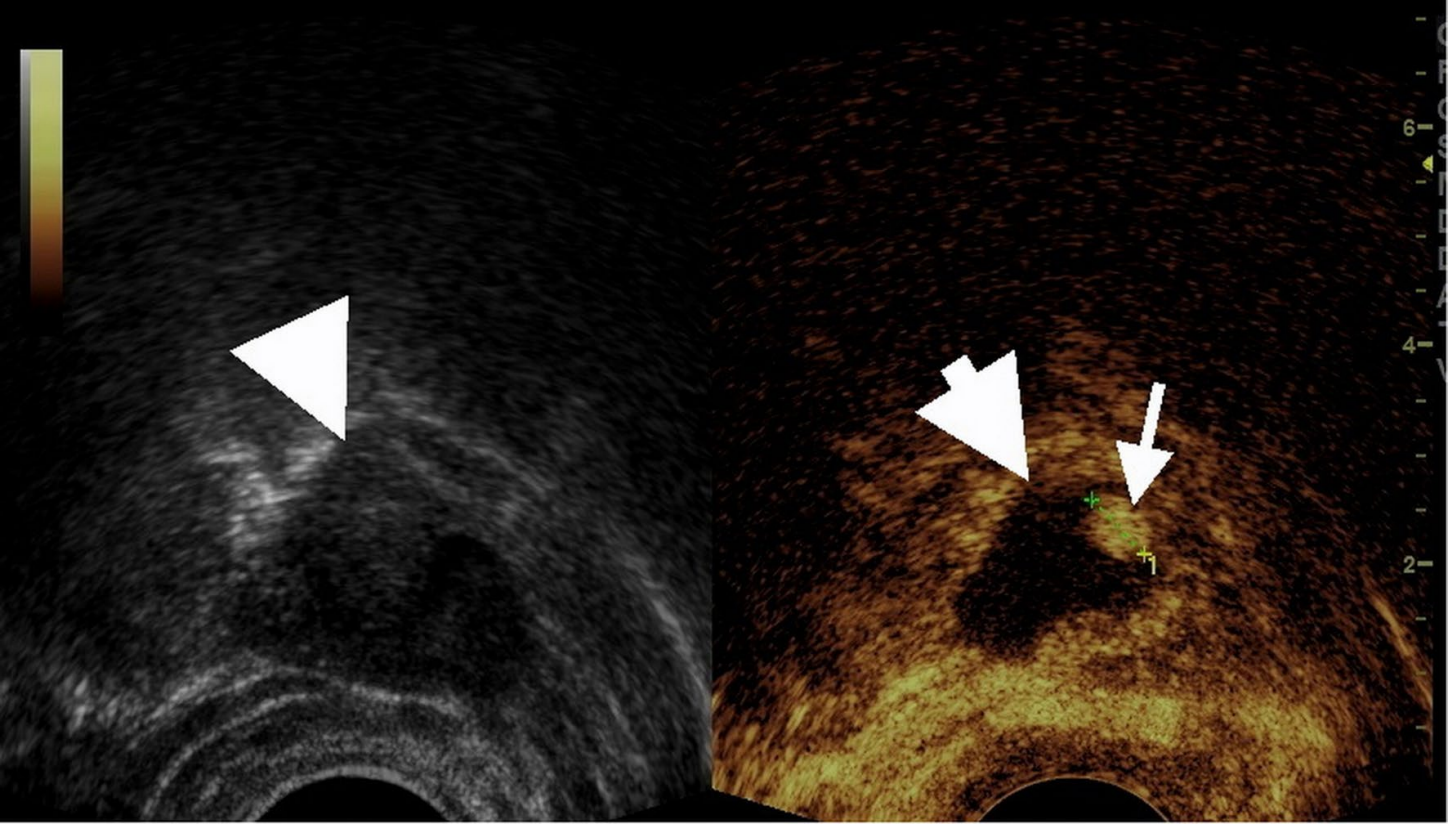
Transvaginal contrast enhanced ultrasound (CEUS) showed a adnexal mass of unknown origin. The patient was a 23-year-old woman who presented with amenorrhea and vaginal bleeding. The patient’s blood HCG level was 1010 mIU/mL, and the time interval between CEUS and blood testing was less than 6 h. A mixed echoic mass measuring 2.4 cm×2.1 cm was seen beside the right ovary (left image). CEUS showed heterogeneous enhancement of the mass. The thick arrow indicated the enhanced cyst wall and the thin arrow indicated the enhanced villous tissue (right image). The ultrasound diagnosis was ectopic pregnancy with the location to be undetermined and the possibility of ovarian pregnancy was relatively high. The final surgical diagnosis confirmed it as a right ovarian pregnancy, with the villous tissue measuring 0.8 cm×0.8 cm, similar to the ultrasound measurement (0.8 cm×0.6 cm). The non-enhanced areas within the lesions were surgically confirmed to be blood clots

The CEUS performance of a patient with ovarian pregnancy was as follows: peripheral cyst wall and internal wall nodular enhancement, with no enhancement in most other areas.The non-enhanced areas were confirmed to be clots, and the nodular enhanced areas were villous tissue by surgical pathology (Figure 5).

**Fig. 6:**
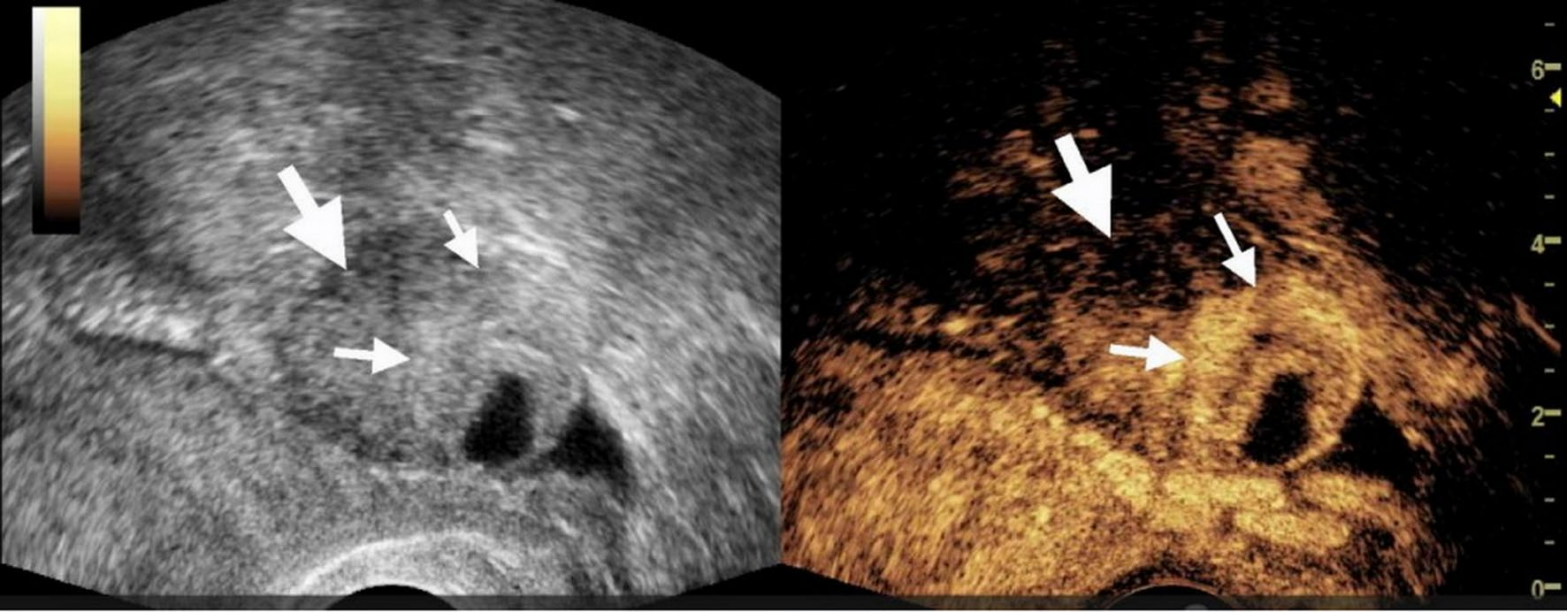
Transvaginal contrast enhanced ultrasound(CEUS) misdiagnosed the luteal structure in the ovary as an adnexal pregnancy. This was a case of intrauterine pregnancy misdiagnosed as ectopic pregnancy by CEUS. The patient, a 28-year-old woman, visited the hospital due to abdominal pain. Her blood HCG level was 84 mIU/mL. The left image showed a high-echo mass (thin arrow) near the left ovary (thick arrow), measuring 2.2cm×2.0cm. The right image of CEUS showed significant enhancement of the mass with a crescent moon shape. The enhancement of the ovary was less than the mass. The ultrasound diagnosis was ectopic pregnancy with the location to be undetermined and the possibility of ovarian pregnancy was relatively high. The patient was later confirmed to have anuterine pregnancy. After a curettage, the patients HCG levels returned to normal and no abnormalities were seen in the ovaries

The CEUS performance of a patient with intrauterine pregnancy was as follows: peripheral cyst wall and internal enhancement with a crescent moon shape, with no enhancement in other areas of the mass.This case of intrauterine pregnancy was misdiagnosed as EP (Figure 6).

### The value of CEUS in diagnosing tEP

Based on positive β-HCG, absence of an intrauterine gestational sac, and sonographic visualization of a dilated fallopian tube containing either circular or non-circular enhancement internally, CEUS demonstrated high diagnostic accuracy for tEP diagnosis in cases with low β-HCG levels. CEUS correctly diagnosed 18 of 19 tEP cases. One tEP case was diagnosed as a mass of uncertain significance. The intrauterine pregnancy case was misdiagnosed as an EP. The ovarian pregnancy case was diagnosed as EP, though CEUS indicated a relatively high possibility of ovarian origin.

## Discussion

Previously, it was believed that a discriminatory serum HCG level was generally between 1500 and 2000 mIU/Ml [31, 32], but this result has been challenged [33, 34].In the study of Peter M Doubilet, among 202 HCG positive patients with no gestational sac in the uterus, 12 patients (5.9%) had HCG levels ranging from 1500 to 1999mIU/mL, and 9 patients (4.5%) had levels greater than 2000 mIU/mL. Finally, all patients were confirmed to have intrauterine pregnancy [33].American College of Obstetricians and Gynecologists recommends that a discriminatory zone should be set at 3500mIU/mL [18]. Therefore, in our study, 3500mIU/mL was used as discriminatory HCG levels. When HCG levels are low, ultrasound is very difficult to distinguish between intrauterine pregnancy, spontaneous miscarriage, and EP. Therefore, we introduced CEUS in our study. Referring to other studies [33, 34], in our research design, It was required that β-HCG level in plasma was lower than 3500 mIU/mL and the time interval between CEUS and β-HCG detection was less than 24 h or β-HCG level in plasma was lower than 500 mIU/mL and the time interval between CEUS and β-HCG detection is 24 to 48 h.In our study, 20 of the 21 patients had an interval of less than 24 h, and only 1 patient had an interval of 24–48 h. Since the patient’s plasma HCG level was only 350mIU/mL, according to the HCG growth pattern of tEP, the patient’s plasma HCG level measured within 24 h must be less than 3500mIU/mL.The study by B G Bateman et al. indicates that the HCG doubling time in tubal pregnancies was 7.69 +/- 9.8 days [35].

Compared with conventional ultrasound, CEUS has higher soft tissue resolution for an adnexal mass. It can clearly distinguish soft tissues from clots, as latter appear nonenhanced. EP patients usually present with adnexal masses [11]. Conventional ultrasound can only provide information on the size, shape, and rough blood of the mass, and more detailed blood supply information cannot be showed. In this study, the sensitivity, specificity, and accuracy of TVUS and CEUS in diagnosing tubal dilation were 15.8%, 100%, 23.8%, and 94.7%, 100%, and 95.2%, respectively.There were statistically significant differences in sensitivity and accuracy between CEUS and TVUS (*P* < 0.05).Besides, Conventional ultrasound can only give a diagnosis of suspected dilation of fallopian tubes, and it cannot clearly distinguish the fallopian tubes from the internal clots, nor can it identify villous issue.Therefore, our study indicated that CEUS was of great value in diagnosing tubal dilation.The mechanism of CEUS for the diagnosis of tubal dilation in patients with tEP is as follows: The tubal wall is a structure that can enhanced because of the blood supply, which appears as a ring structure that can be enhanced in the transverse section and as a tubular structure that can be enhanced in the longitudinal section. Inside of the fallopian tube is often filled with blood clots, which is a structure without blood supply.So, blood clots is a non-enhanced tissue, and the dilated fallopian tube can be clearly displayed by CEUS. However, there is little difference in echogenicity between the blood clot and the tubal wall, so it is difficult to clearly display the internal structure of the fallopian tube by TVUS.

There were only a few literatures on the use of CEUS in the diagnosis of EP, and they mainly focus on scar or cervical pregnancy.These studies have found that EP generally presents as local high enhancement [26–29]. In one article, three cases were reported including an intramural pregnancy after hysteromyomectomy, and two mass-based cesarean scar pregnancy [29]. In these cases, early enhancement with high intensity was observed by CEUS at the site of implantation, moreover, prominently enhanced signal was detected inside the lesions, with or without peritrophoblastic ring [29]. One article showed that using a cut-off value of 1.08 for the peak intensity ratio of caesarean scar to myometrium, the diagnosis sensitivity, specificity, positive predictive value and negative predictive value for caesarean scar pregnancy were 77.8, 100, 100, and 81.8%, respectively [28].

According to its ultrasound characteristics, enhancement pattern of villous tissue was divided into two types: circular enhancement and non-circular enhancement.In our study, the two cases with circular enhancement had HCG values greater than 900 mIU/mL, while the median HCG value for non-circular enhancement was 498 mIU/mL. Different enhancement patterns may be related to the stage of disease progression, with non-circular enhancement indicating abortion of the EP and ring enhancement indicating an unrupt gestational sac. The mechanism of CEUS in the diagnosis of intrafallopian chorionic tissue is as follows: It is well known that chorionic tissue is a highly vascularized tissue, so in the application of CEUS, an enhanced soft tissue structure adjacent to the wall of the fallopian tube can be found.It is the basic sign of CEUS in the diagnosis of intrafallopian villous tissue.In contrast to the ovaries, tubal intraluminal mass-like enhancement is rare, and thus, the accuracy of CEUS in the diagnosis of villous tissue within the fallopian tubes of patients with tEP is also high.Interestingly, the bagel sign was seen in one patient on conventional ultrasound, while CEUS showed non-ring enhancement which was showed in Fig. 3. Some areas with high echogenic were considered villous tissue on conventional ultrasound, but CEUS suggested blood clots, which was confirmed by surgery. Therefore, the bagel sign on conventional ultrasound was not reliable for diagnosing villous tissue.

Our article showed that based on the following evidence: positive HCG levels, absence of an intrauterine gestational sac, and sonographic visualization of dilated fallopian tubes containing circular or non-circular enhancement inside, CEUS had a high accuracy rate in definitive diagnosis of tEP with low level of HCG. The accuracy of CEUS diagnosis was as follows: 18 cases of tEP were correctly diagnosed(18/19).Due to the absence of misdiagnosis case, CEUS had a strong specificity in the diagnosis of tubal pregnancies. However, due to the small sample size, this study did not analyze the sensitivity, specificity, and accuracy of CEUS in the diagnosis of tubal pregnancies.

One case of intrauterine pregnancy was misdiagnosed as EP in our study, because we mistook the enhanced structure with a crescent moon shape for villous tissue.This misdiagnosis was related to our insufficient early diagnostic experience. It can be seen from the enhanced performance that the size of the enhanced nodule was approximately 2.2cmx2.0 cm, while the patient’s plasma HCG level was only 84mIU/mL, which was not matched. However, if the HCG level of this patient was relative high (such as HCG was 1000-3000mIU/),diagnosis will become very difficult.There were some diagnostic pitfalls: (1) The ring-like enhancement of the peripheral wall was prone to be misdiagnosed as fallopian tube wall; (2) The crescent enhancement was prone to be misdiagnosed as the villous tissue. We were confident in the diagnosis of tEP because of the clear display of the internal structure of the fallopian tube by CEUS; however, we lacked confidence in the differential diagnosis of intraovarian pregnancies and other structures such as corpus luteum. In a word, for a part of intrauterine pregnancy patients, CEUS may lead to a false-positive diagnosis of EP.Therefore, the application of CEUS in diagnosing ovarian pregnancies should be cautious, and other imaging examinations should be applied when necessary.This depends on the accumulation and summary of experience in the future.

Our research suggests that positive HCG levels, absence of an intrauterine gestational sac, dilated fallopian tubes combined with enhanced embryonic villous tissue within the fallopian wall can serve as clear diagnostic indicators for EP in the fallopian tubes with low level β-HCG (β-HCG level in plasma was lower than 3500 mIU/mL).We suggest that CEUS can be used as a method for further examination of EP when conditions permit.

In terms of safety, since CEUS is generally not used in pregnant women, there were few related studies. In the literature on the application of CEUS in EP, no adverse reactions have been reported.In a study, CEUS was safely performed on six pregnant women without the occurrence of adverse fetal or maternal events(mean weeks of pregnancy: 28 weeks) [36].

Limitations of this study include: this study was a retrospective analysis; the sample size of the study was small; it was a single-center study.Due to the existence of these research limitations, the following situations occur: (1) Due to the single-center source of patients, the research results have regional characteristics.When the research results are generalized to a broader range, they need to be carefully verified; (2) Due to the small sample size, the random sampling bias may be large; (3) Compared with prospective studies, retrospective studies are inferior in terms of data quality and integrity, recall bias, and the influence of unknown confounding factors. In future, a prospective, multi-center study with cost-effectiveness analysis will be conducted. Since no related studies have been published before, this study, despite having many shortcomings, can open the horizons of radiologists and clinicians.

## Conclusions

In conclusion, CEUS holds significant diagnostic value for tEP. It is particularly useful in diagnostically unclear cases and provides a more detailed assessment of the internal structure of adnexal masses.

## Data Availability

The datasets used and/or analysed during the current study are available from the corresponding author on reasonable request.

## Abbreviations

EP: Ectopic pregnancy
TVUS: Transvaginal ultrasound
HCG: Human chorionic gonadotropin
CEUS: Contrast enhanced ultrasound
